# Clinicopathological characteristics and prognosis of metaplastic breast cancer versus triple-negative invasive ductal carcinoma: a retrospective analysis

**DOI:** 10.1186/s12957-023-03261-w

**Published:** 2023-11-24

**Authors:** Xiaolu Yang, Tiantian Tang, Tao Zhou

**Affiliations:** 1 Hebei Medical University, Shijiazhuang, China; 2 Department of Breast Cancer Center, The Fourth Hospital of Hebei Medical University, No. 12, Jian-Kang Road, Shijiazhuang, 050011 China

**Keywords:** Metaplastic breast cancer, Triple-negative invasive ductal carcinoma, Clinicopathological characteristics, Prognosis

## Abstract

**Background:**

Metaplastic breast cancer(MBC) is a specific pathological type of invasive breast cancer. There are few studies related to MBC due to its rarity. This study aimed to analyse the differences in clinicopathological characteristics and prognosis between Metaplastic breast cancer and triple-negative invasive ductal carcinoma (TN-IDC).

**Methods:**

We retrospectively compared the clinicopathological characteristics of patients diagnosed with MBC and TN-IDC at the Fourth Hospital of Hebei Medical University between 2011 and 2020 in a 1:2 ratio. The log-rank test was used to compare the two groups’ disease-free survival (DFS) and overall survival (OS). For MBCs, we performed univariate and multivariate analyses using the Cox proportional hazards model to determine the characteristics that impacted OS and DFS.

**Results:**

A total of 81 patients with MBC and 162 patients with TN-IDC were included in this study. At initial diagnosis, MBC patients had larger tumour diameters(*P* = 0.03) and fewer positive lymph nodes (*P* = 0.04). Patients with MBC were more likely to have organ metastases after surgery (*P* = 0.03). Despite receiving the same treatment, MBC patients had worse DFS (HR = 1.66, 95%CI 0.90–3.08, *P* = 0.11) and OS (HR = 1.98, 95% CI 1.03–3.81, *P* = 0.04), and OS was statistically significant. Positive lymph nodes at initial diagnosis were associated with worse DFS (HR = 3.98, 95%CI 1.05–15.12, *P* = 0.04) and OS (HR = 3.70, 95%CI 1.03–13.34, *P* = 0.04) for patients with MBC. The efficacy of platinum-based agents is insensitive for MBC patients receiving chemotherapy. In addition, patients treated with preoperative chemotherapy had worse DFS compared to patients treated with postoperative chemotherapy (HR = 3.51, 95%CI 1.05–11.75, *P* = 0.04).

**Conclusions:**

The clinicopathological characteristics and prognosis of MBC and TN-IDC differ in many ways. Further studies are required to determine suitable treatment guidelines for patients with MBC.

## Background

Metaplastic breast cancer (MBC) is a rare and clinically distinct type of breast cancer with diverse pathological characteristics. It is a poorly differentiated, heterogeneous tumour originating from epithelial and mesenchymal cells [1]. The World Health Organization categorises MBC based on tumour pathology as squamous cell carcinoma, low-grade adenosquamous carcinoma, metaplastic carcinoma with mesenchymal differentiation, spindle cell carcinoma, fibromatosis-like metaplastic carcinoma, mixed metaplastic carcinoma, and myoepithelial carcinoma [2].

There are relatively few studies on MBC, as first reported by Huvos [3] in 1973; however, it was not until 2000 that MBC was considered a separate pathological type. According to available studies, the immunohistochemical expression of MBC suggests that it is generally triple-negative, indicating that it is negative for both the estrogen receptor (ER) and progesterone receptor (PR) and does not overexpress human epidermal growth factor receptor-2 (HER-2) [4, 5]. Consequently, many specialists regard MBC as a subtype of triple-negative breast cancer (TNBC) [6, 7]. Owing to the low incidence and rarity of MBC, this subtype is poorly understood and has no established clinical treatment guidelines. The National Comprehensive Cancer Network (NCCN) suggests that patients with MBC should be treated according to the guidelines for invasive ductal carcinoma (IDC) because it is thought to have the same prognosis [8]. Although experts recommend referencing IDC for the management of MBC, this does not imply that the clinicopathological features of MBC and IDC are identical [9]. Moreover, the prognoses of MBC and IDC may differ, even when the same treatment is employed [10].

This study aimed to investigate the clinicopathological and prognostic characteristics of MBC and the factors influencing the prognosis of patients with MBC. We compared patients with MBC and those with triple-negative IDC (TN-IDC) to accomplish our goal. Further studies of the MBC population were undertaken to investigate the factors affecting prognosis.

## Methods

### Study design and patients

We retrospectively reviewed 84 patients with surgically pathologically confirmed MBC treated at the Fourth Hospital of Hebei Medical University between January 2011 and December 2020. We excluded patients with incomplete medical records, cancers of other organs, and bilateral breast cancer. A total of 81 patients with MBC were included in this study. We reviewed 1666 TN-IDC patients and randomly selected 162 TN-IDC patients in a 1:2 ratio for comparison with MBC patients using a propensity-score matched analysis based on time to surgery.

We obtained and analysed the following variables: patient’s age at the time of diagnosis, menstrual status, tumour size, lymph node (LN) status, distant metastasis, AJCC stage, biomarker profiles (ER, PR, and HER2) of the tumour, therapy modality (surgery, chemotherapy regimen, and radiotherapy), and survival data including disease-free survival (DFS) and overall survival (OS).

DFS was defined as the time from the date of surgical excision of the tumour to the date of disease recurrence (including distant metastasis and local recurrence) or death from any cause, whereas OS was defined as the time from the diagnosis of the disease to the patient’s death from any cause. The last follow-up period was used for patients lost to follow-up before recurrence or death.

### Statistical analysis

The* t* test and *x*^*2*^ test were used to compare the clinicopathological characteristics between the two groups. The Wilcoxon test was used to compare the grade data. DFS and OS rates were calculated using the Kaplan–Meier method, and comparisons were made between groups using the log-rank test. For patients with MBC, we performed univariate and multivariate analyses using the Cox proportional hazards model to determine the characteristics that had an impact on OS and DFS. Statistical significance was defined as a *p value* < 0.05. SPSS software (version 25.0) was used for the statistical analysis.

## Results

### Clinicopathological and treatment characteristics

We included 81 and 162 patients with MBC and TNBC, respectively. Among the 81 patients with MBC, 68 were triple-negative, seven were HR( −) and HER-2( +), and six were HR( +) and HER-2( −). Table 1 shows the clinicopathological and treatment characteristics of the two groups. The mean ages of the patients with MBC and TN-IDC were 51.07 and 52.35 years, respectively. There were no statistically significant differences in mean age between the two groups at the time of diagnosis (*P* = 0.41), nor were there any statistically significant differences in menstrual status between the two groups at the time of diagnosis (*P* = 0.20).

**Table 1:** Comparison of clinicopathological features of patients in MBC and TN-IDC

	MBC *N* = 81	TN-IDC *N* = 162	*P* value
**Age**			0.41
Mean age (year)	51.07 ± 1.291	52.35 ± 0.876	
**Age**			0.93
≤ 50	38(47%)	75(46%)	
> 50	43(53%)	87(54%)	
**Menstrual state**			0.20
Premenopausal	44(54%)	74(46%)	
Postmenopausal	37(46%)	88(54%)	
**Tumour size(cm)**			0.03
≤ 2	23(28%)	74(46%)	
> 2	50(62%)	78(48%)	
Unknown	8(10%)	10(6%)	
***T* stage**			0.04
TX	8(10%)	10(6%)	
T1	24(30%)	73(45%)	
T2	41(50%)	65(40%)	
T3	4(5%)	6(4%)	
T4	4(5%)	8(5%)	
**LN status**			0.04
Negative	60(74%)	98(60%)	
Positive	21(26%)	64(40%)	
***N* stage**			0.03
N0	60(74%)	98(60%)	
N1	10(12%)	30(19%)	
N2	7(9%)	14(9%)	
N3	4(5%)	20(12%)	
**Distant metastasis**			1.00
No	79(98%)	159(98%)	
Yes	2(2%)	3(2%)	
**AJCC stage**			0.58
I	20(25%)	56(35%)	
II	43(53%)	62(38%)	
III	10(13%)	31(19%)	
IV	2(2%)	3(2%)	
Unknown	6(7%)	10(6%)	
**AJCC stage**			0.20
I+II	63	118	
III	10	31	
**Subtype**			–
Triple negative	68(84%)	162	
HR( −) HER-2( +)	7(9%)	0	
HR( +) HER-2( −)	6(7%)	0	
**Chemotherapy**			0.12
Yes	72(89%)	153(94%)	
No	9(11%)	9(6%)	
**Chemotherapy modalities**			0.29
Postoperative chemotherapy	55(76%)	126(82%)	
Preoperative chemotherapy	17(24%)	27(18%)	
**Radiotherapy**			0.38
Yes	41(51%)	70(43%)	
No	35(43%)	85(53%)	
Unknown	5(6%)	7(4%)	
**Endocrine therapy**			–
Yes	5(6%)	0(0)	
No	76(94%)	162(100%)	
**Anthracycline therapy**			0.62
Yes	64(89%)	133(87%)	
No	6(8%)	16(10%)	
Unknown	2(3%)	4(3%)	
**Taxane therapy**			0.41
Yes	64(89%)	142(93%)	
No	6(8%)	7(5%)	
Unknown	2(3%)	4(2%)	
**Platinum therapy**			0.26
Yes	9(13%)	12(8%)	
No	61(85%)	137(90%)	
Unknown	2(2%)	4(2%)	
**Operation**			0.91
Mastectomy	66(81%)	133(82%)	
BCS	15(19%)	29(18%)	
**Operation**			0.41
ALND	42(52%)	93(57%)	
SLNB	39(48%)	69(43%)	

*MBC* metaplastic breast cancer, *TN-IDC* triple-negative invasive ductal carcinoma, *T* tumour size, *LN* lymph node, *N* nodal size, *AJCC* American Joint Committee on Cancer, *HR* hormone receptor, *HER-2* human epidermal growth factor receptor-2, *BCS* breast-conserving surgery, *ALND* axillary lymph node dissection, *SLNB* sentinel lymph node biopsy

We found that 50 patients (62%) in the MBC group and 78 patients (48%) in the TN-IDC group had tumours > 2 cm in diameter. Sixty (74%) patients in the MBC group were lymph node-negative, while 98 (60%) patients in the TN-IDC group were lymph node-negative. Compared to the TN-IDC group, MBC patients had larger tumours (*P* = 0.03) and fewer lymph node metastases (*P* = 0.04). The distributions of the *T* stage (*P* = 0.04) and *N* stage (*P* = 0.03) were also statistically different between the two groups, but there was no statistical difference in distant metastasis (*P* = 1.00) or AJCC stage (*P* = 0.58).

There was no difference in the proportion of persons in the two groups who chose breast-conserving surgery (BCS) (19% vs. 18%, *P* = 0.91) nor in the proportion who chose sentinel lymph node biopsy (SLNB) (48% vs. 43%, *P* = 0.41). Patients in both groups were more likely to undergo mastectomy and axillary lymph node dissection (ALND).

Seventy-two patients (89%) in the MBC group and 153 (94%) in the TN-IDC group were treated with chemotherapy, with no significant difference between the two groups. Both groups were more likely to choose postoperative chemotherapy (76% vs. 82%, *P* = 0.29). Both groups favoured anthracycline chemotherapy drugs (89% vs. 87%, *P* = 0.62) and paclitaxel chemotherapy drugs (89% vs. 93%, *P* = 0.41) over platinum-based drugs (13% vs. 8%, *P* = 0.26) when selecting a chemotherapy regimen. No statistical difference in the chemotherapy modality or regimen between MBC and TN-IDC existed. There was also no statistically significant difference in the proportion of patients receiving radiotherapy between the two groups.

### Survival results for MBC vs. TN-IDC

**Fig. 1:**
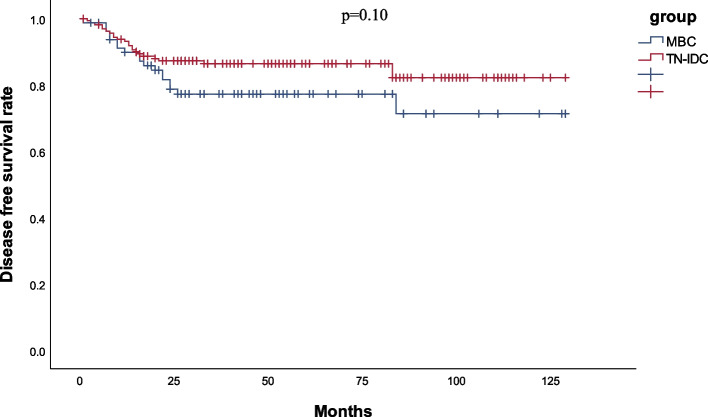
Disease-free survival curves of MBC and TN-IDC patients

**Fig. 2:**
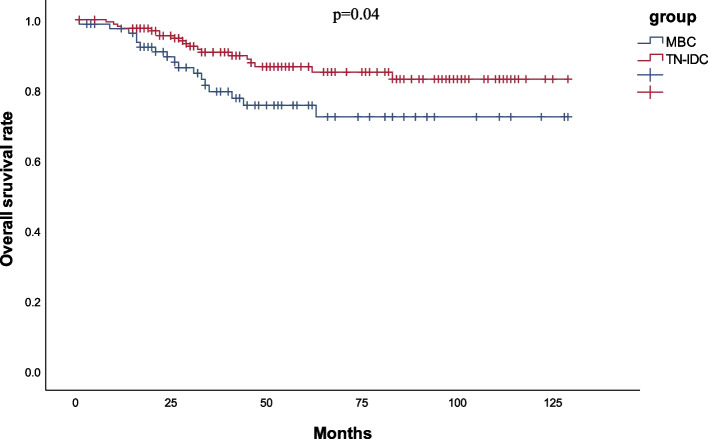
Overall survival curves of MBC and TN-IDC patients

The median follow-up periods were 52 and 53 months for the MBC and TN-IDC groups, respectively. DFS and OS were evaluated using a Kaplan–Meier plot to compare prognoses between the groups. Figures 1 and 2 show the survival curves of DFS and OS for MBC versus TN-IDC. The 5-year DFS rate was 77.3% in the MBC group and 86.4% in the TN-IDC group (*P* = 0.10). The 5-year OS rate was 75.6% in the MBC group and 86.6% in the TN-IDC group (*P* = 0.04). The OS of patients with MBC was significantly worse than that of patients with TN-IDC, with hazard ratio (HR) ratios of 1.98 (95% CI 1.03–3.81, *p* = 0.04). DFS was also worse in the MBC group than the TN-IDC group, though the difference was insignificant (HR = 1.66,95%CI 0.90–3.08, *P* = 0.11).

The postoperative characteristics of the two populations are shown in Table 2. Eighteen of the 81 patients in the MBC group experienced disease recurrence, and 17 patients died. Twenty-three of the 162 patients in the TN-IDC group experienced disease recurrence, and 19 died. The incidences of recurrence and death did not differ significantly between the two groups. However, we discovered that 17 patients (21%) in the MBC group had organ metastases during follow-up compared to 17 patients (10%) in the TN-IDC group, who were more likely to experience organ metastatic events in the MBC group than those in the TN-IDC group (*P* = 0.03). The most prevalent site of metastasis in the MBC group was the lungs (15% vs. 5%, *P* = 0.01), followed by bones (9% vs. 2%, *P* = 0.03).

**Table 2:** Clinical course comparison of MBC and TN-IDC groups during follow-up

	MBC (*n* = 81)	TN-IDC (*n* = 162)	*P*
**Recurrence**			0.11
( +)	18(22%)	23(14%)	
( −)	63(78%)	139(86%)	
**Death**			0.05
( +)	17(21%)	19(12%)	
( −)	64(79%)	143(88%)	
**Local recurrence**			1
( +)	1(1%)	3(2%)	
( −)	80(99%)	159(98%)	
**Organ metastasis**			0.03
( +)	17(21%)	17(10%)	
( −)	64(79%)	145(90%)	
**Brain metastasis**			0.91
( +)	3(4%)	8(5%)	
( −)	78(96%)	154(95%)	
**Lung metastasis**			0.008
( +)	12(15%)	8(5%)	
( −)	69(85%)	154(95%)	
**Liver metastasis**			1
( +)	4(5%)	8(5%)	
( −)	77(95%)	154(95%)	
**Bone metastasis**			0.03
( +)	7(9%)	3(2%)	
( −)	74(91%)	159(98%)	
**Bone marrow metastasis**			1
( +)	1(1%)	1(1%)	
( −)	80(99%)	161(99%)	
**Pleural metastasis**			
( +)	2(3%)	0	
( −)	79(97%)	162	

### Univariate and multivariate analyses of DFS and OS of MBC

We conducted univariate and multivariate analyses using Cox proportional hazards models to further investigate the characteristics affecting DFS and OS in patients with MBC. The results are shown in Tables 3 and 4. Among the variables evaluated for DFS, higher *T* stage (T3 + T4) (HR = 4.37, 95%CI 1.39–13.69, *P* = 0.01), lymph node positivity (HR = 7.40, 95%CI 2.77–19.82, *P* < 0.01), distant metastasis (HR = 12.48, 95%CI 2.62–59.44, *P* < 0.01), and higher AJCC stage (III + IV) (HR = 7.83, 95%CI 3.07–19.95, *P* < 0.01) were significantly associated with worse DFS. These variables were also significantly associated with worse OS (*T* stage, HR = 5.37, 95%CI 1.80–19.71, *P* < 0.01; lymph node positivity, HR = 6.15, 95%CI 2.27–16.67, *P* < 0.01; distant metastasis HR = 10.53, 95%CI 2.18–50.85, *P* < 0.01; higher AJCC stage, HR = 6.04, 95%CI 2.32–15.73, *P* < 0.01). The results of the multivariate analysis indicated that lymph node-positive was the only factor associated with worse DFS (HR = 3.98, 95%CI 1.05–15.12, *P* = 0.04) and OS (HR = 3.70, 95%CI 1.03–13.34, *P* = 0.04). In addition, we found that age, menstrual status, mode of surgery, chemotherapy, and radiotherapy did not affect DFS or OS.

**Table 3:** Univariate and multivariate analyses of DFS in MBC

	DFS			
	Univariate analysis		Multivariate analysis	
	HR	*P* value	HR	*P* value
Age ≤ 50	1.57(0.62–4.00)	0.34		
Premenopausal	1.14(0.49–2.88)	0.79		
T(T3 + T4)	4.37(1.39–13.69)	0.01	1.49(0.31–7.11)	0.62
LN(positive)	7.40(2.77–19.82)	< 0.01	3.98(1.05–15.12)	0.04
M(M1)	12.48(2.62–59.44)	< 0.01	2.19(0.30–16.25)	0.44
AJCC stage(III + IV)	7.83(3.07–19.95)	< 0.01	2.30(0.61–8.69)	0.22
Subtype(TN-MBC)	0.60(0.20–1.83)	0.37		
Chemotherapy (yes)	2.29(0.30–17.15)	0.42		
Radiotherapy (yes)	2.38(0.88–6.45)	0.88		
Endocrine therapy (yes)	0.95(0.13–7.18)	0.96		
Operation (BCS)	0.55(0.13–2.40)	0.43		
Operation (SLNB)	0.52(0.19–1.41)	0.20		

*DFS* disease-free survival, *MBC* metaplastic breast cancer, *HR* hazard ratio, *T* tumour size, *LN* lymph node, *M* distant metastasis at diagnosis, *TN-MBC* triple-negative metaplastic breast cancer, *AJCC* American Joint Committee on Cancer, *BCS* breast-conserving surgery, *SLNB* sentinel lymph node biopsy

**Table 4:** Univariate and multivariate analyses of OS in MBC

	OS			
	Univariate analysis		Multivariate analysis	
	HR	*P* value	HR	*P* value
Age ≤ 50	1.48(0.57–3.83)	0.42		
Premenopausal	1.03(0.40–2.67)	0.96		
T(T3 + T4)	5.37(1.80–19.71)	< 0.01	2.39(0.48–11.95)	0.29
LN(positive)	6.15(2.27–16.67)	< 0.01	3.70(1.03–13.34)	0.04
M(M1)	10.53(2.18–50.85)	< 0.01	1.42(0.19–10.50)	0.73
AJCC stage(III + IV)	6.04(2.32–15.73)	< 0.01	1.91(0.51–7.17)	0.34
Subtype(TN-MBC)	0.50(0.16–1.54)	0.22		
Chemotherapy (yes)	1.78(0.24–13.56)	0.57		
Radiotherapy (yes)	1.96(0.71–5.40)	0.19		
endocrine therapy (yes)	1.25(0.17–9.53)	0.83		
Operation (BCS)	0.63(0.14–2.74)	0.53		
Operation (SLNB)	0.71(0.26–1.95)	0.51		

*OS* overall survival, *MBC* metaplastic breast cancer, *HR* hazard ratio, *T* tumour size, *LN* lymph node, *M* distant metastasis at diagnosis, *TN-MBC* triple-negative metaplastic breast cancer, *AJCC* American Joint Committee on Cancer, *BCS* breast-conserving surgery, *SLNB* sentinel lymph node biopsy

We further performed univariate and multivariate analyses of patients with MBC receiving chemotherapy, and the results are shown in Tables 5 and 6. Univariate analysis suggested that preoperative chemotherapy and the use of platinum-based drugs were associated with worse DFS and OS. The multivariate analysis results suggested that patients treated with preoperative chemotherapy tended to suggest worse DFS (HR = 3.51, 95%CI 1.05–11.75, *P* = 0.04), but did not affect OS (HR = 3.33, 95%CI 0.97–11.39, *P* = 0.06).

**Table 5:** Univariate and multivariate analyses of DFS in MBC patients receiving chemotherapy

	DFS			
	Univariate analysis		Multivariate analysis	
	HR	*P* value	HR	*P* value
Preoperative chemotherapy	4.93(1.88–12.89)	< 0.01	3.51(1.05–11.75)	0.04
Anthracycline therapy	1.31(0.17–9.88)	0.79		
Taxane therapy	0.73(0.16–3.24)	0.68		
Platinum therapy	5.04(1.73–14.69)	< 0.01	1.89(0.50–7.18)	0.35

**Table 6:** Univariate and multivariate analyses of OS in MBC patients receiving chemotherapy

	OS			
	Univariate analysis		Multivariate analysis	
	HR	*P* value	HR	*P* value
Preoperative chemotherapy	4.70(1.75–12.63)	< 0.01	3.33(0.97–11.39)	0.06
Anthracycline therapy	1.48(0.20–11.23)	0.70		
Taxane therapy	0.72(0.16–3.16)	0.66		
Platinum therapy	4.84(1.66–14.14)	< 0.01	1.95(0.51–7.35)	0.33

*DFS* disease-free survival, *HR* hazard ratio, *OS* overall survival, *HR* hazard ratio

## Discussion

MBC is a clinically rare breast cancer subtype, accounting for < 1% of all breast cancers [11]. Few studies have been conducted on MBC, and there are currently no treatment guidelines for patients with MBC. When treating patients with MBC, physicians usually turn to the treatment guidelines for IDC. We performed a retrospective analysis of the clinicopathological and prognostic characteristics of patients with MBC and TN-IDC and discovered numerous differences between the two types of cancer. Patients with MBC have unique characteristics.

TNBC accounts for approximately 15% of all subtypes of breast cancer [12]. In our study, 68 of the 81 MBC patients were found to be triple-negative, representing approximately 84%, which suggests that MBC does often present as triple-negative. However, it is inaccurate to assume that MBC is a specific type of TNBC.

Li [13] found that, compared to TNBC patients, MBC patients were often older than 50 years at the time of diagnosis. We found that the mean age at diagnosis was 51.07 years for MBC patients and 52.35 years for TNBC patients, with no difference in age between the two groups. There was also no difference in the proportion of the two groups aged over 50 years, which is consistent with the findings of Aydiner [8].

We found patients with MBC tended to have larger tumour diameters than did those with TN-IDC. Previous studies have demonstrated that patients with larger tumour diameters are more likely to develop lymph node metastases [14]. This is understandable because larger tumour diameters are commonly believed to be associated with more aggressive and poorly differentiated tumours, which are typically associated with lymph node metastasis. However, we observed few lymph node metastases in the MBC group despite the larger tumour diameter. Many other studies have also found that patients with MBC are less likely to have positive lymph nodes than patients with other types of breast cancer [9, 13, 15]. This is an interesting phenomenon, and many experts have speculated that MBCs tend to develop haematogenous rather than lymphatic metastases, which is a unique pathologic feature of MBC [16, 17]. However, our study did not find an increased incidence of distant metastases among patients with MBC at the time of the initial diagnosis, which may be related to the small sample size.

Although we did not find a higher probability of haematogenous metastases in patients with MBC at the time of initial diagnosis, we found that 21% of patients with MBC developed organ metastases after surgery, compared to 10% of patients with TN-IDC. Compared with the TN-IDC group, the MBC group was more likely to develop organ metastases postoperatively, supporting the notion that MBC is more likely to metastasise via the bloodstream. When analysing the metastatic sites, we discovered that the lungs were the most common site of metastasis in the MBC group, followed by the bone, which is consistent with the findings of Mckinnon [18].

We found no differences in the surgical procedures, chemotherapy, chemotherapeutic modalities, regimens, or radiotherapy employed between the two groups when comparing their respective treatments. This also indicates that MBC patients were seen as IDC patients for treatment. Nevertheless, the MBC group had a worse OS despite receiving identical treatments. There are two possible explanations for this: first, MBC is a more malignant tumour, and the prognosis for MBC patients tends to be worse; second, the treatment guidelines for IDC patients may not be appropriate for MBC patients. Therefore, we expect that additional studies will be conducted to identify more effective treatments for MBC.

In this study, both DFS and OS were worse in the MBC group than in the TN-IDC group, with a statistically significant difference in OS. We performed univariate and multivariate analyses to analyse the factors affecting the prognosis of patients with MBC. Univariate analysis showed that higher *T* stage (T3 + T4), lymph node positivity, distant metastasis, and higher AJCC stage (III + IV) were associated with worse DFS and OS. Multivariate analysis suggested that only patients with positive lymph nodes at initial diagnosis had a worse prognosis. By comparing the clinicopathological features, we found that MBC did not often metastasize through the lymph nodes; however, once a patient with MBC has metastases in the lymph nodes, this is a sign of high disease malignancy and often represents a worse prognosis.

According to previous studies, patients with MBC who undergo breast-conserving surgery have better prognoses than those who undergo mastectomy [19, 20]. However, this study found that the two surgical procedures did not affect the prognosis of the patients, which is different from the results of existing studies. Prospective or retrospective studies with larger sample sizes are needed to determine whether the surgical approach influences MBC patients’ survival.

Most patients with MBC choose to undergo chemotherapy; however, chemotherapy has not improved the prognosis of patients with MBC. Multiple studies have also found low sensitivity of MBC to chemotherapy, both preoperative and postoperative [21–23]. Multivariate analysis of patients with MBC who received chemotherapy revealed that preoperative chemotherapy was associated with lower DFS. Chen et al. [24] found that the probability of achieving a pathologic complete response (pCR) after preoperative chemotherapy in patients with MBC was low at 10%. This suggests that MBC is not sensitive to chemotherapy and that preoperative chemotherapy is not recommended for patients. Patients with MBC who undergo preoperative chemotherapy may experience a worsening of their disease, making surgery more challenging and reducing their chances of survival. Until now, only two studies have reported the effect of different chemotherapeutic agents on the prognosis of patients with MBC [8, 25]. Consistent with the study by Aydiner [8], we discovered that anthracycline and paclitaxel were the most commonly used drugs in patients with MBC. We found that neither paclitaxel nor anthracycline improved the prognosis of the patients, in contrast to Aydiner’s findings that paclitaxel improved the OS of patients. Similarly, Morgan [25] concluded that paclitaxel had no effect on the prognosis of MBC patients in terms of DFS or OS. By univariate analysis, we found that platinum-based drugs were associated with poorer DFS and OS. Therefore, regarding the choice of chemotherapy regimen, we do not recommend the use of platinum-based drugs. Although anthracyclines and paclitaxel have also failed to improve the prognosis of patients with MBC, there may be no better option.

Our study also has limitations. First, this research is single-centre, and the cases included may have local characteristics and limitations. Secondly, selection bias is inevitable because of the retrospective study. In addition to this, the sample size included in this study was small, and conclusions from an expanded sample size would have been more reliable. Due to the limited sample size, we did not analyse MBC into subtypes. However, our study has been one of the largest single-centre retrospective studies with the largest sample size to date, and it’s representative and credible due to the long follow-up period.

## Conclusions

Comparing 81 patients with MBC and 162 patients with TN-IDC, we discovered that patients with MBC had a larger tumour diameter but a lower likelihood of being lymph node-positive. Patients with MBC are more likely to develop organ metastases after surgery. MBC is more likely to have haematogenous metastases than lymph node metastases. Patients with MBC had worse DFS and OS than those with IN-IDC, with OS being significant. Platinum-based medications are ineffective in patients with MBC receiving chemotherapy, and preoperative chemotherapy is associated with lower DFS; therefore, preoperative chemotherapy is not recommended.

## Data Availability

Anyone who wants to get the data can contact email 13930462390@163.com.

## Abbreviations

AJCC: American Joint Committee on Cancer
ALND: Axillary lymph node dissection
BCS: Breast-conserving surgery
CI: Confidence interval
DFS: Disease-free survival
ER: Estrogen receptor
HER-2: Human epidermal growth factor receptor-2
HR: Hazard ratio
HR(-): Hormone receptor-negative
HR( +): Hormone receptor-positive
IDC: Invasive ductal carcinoma
LN: Lymph node
MBC: Metaplastic breast cancer
OS: Overall survival
pCR: Pathologic complete response
PR: Progesterone receptor
SLNB: Sentinel lymph node biopsy
TNBC: Triple-negative breast cancer
TN-IDC: Triple-negative invasive ductal carcinoma
